# Assessment of potentially inappropriate medications using the EU (7)-PIM list and the Swedish quality indicators

**DOI:** 10.1007/s11096-019-00847-x

**Published:** 2019-06-10

**Authors:** Natacha Wamil, Sofia Mattsson, Maria Gustafsson

**Affiliations:** Department of Pharmacology and Clinical Neuroscience, Umeå University, 901 87 Umeå, Sweden

**Keywords:** Aged, EU (7)-PIM list, Potentially inappropriate medication, Quality indicators, Sweden

## Abstract

*Background* Several tools to evaluate the appropriateness of prescriptions have been developed over the years. *Objective* To compare the prevalence of potentially inappropriate medication (PIM) among elderly, using the European Union (EU) (7)-PIM list and the Swedish quality indicators. Secondary objectives were to investigate factors associated with the use of PIMs using the two tools. *Setting* Medical ward in a hospital in Northern Sweden. *Methods* Medical records for patients aged ≥ 65 years admitted to the medical ward were reviewed by clinical pharmacists from September to November 2015 and from February to April 2016. PIMs were identified through the abovementioned identification tools. *Main outcome measure* Prevalence of PIMs. *Results* Of 93 patients, 18.3% had one PIM according to the Swedish quality indicators. The most common PIM class was non-steroidal anti-inflammatory drugs and diclofenac was one of the most commonly prescribed PIMs. According to the EU (7)-PIM list, 45.2% of the study population was prescribed one or more PIMs. The most common PIM class was hypnotic and sedative drugs, and the most frequently prescribed PIM was apixaban. No significant associations between PIMs and different factors were found using either identification tool. *Conclusion* The prevalence of PIMs was relatively low in the study sample according to the Swedish guidelines but high according to the EU (7)-PIM list. Different evaluation tools might give inconclusive results, but it is still important to continuously evaluate the need for PIMs in older patients in order to improve drug treatment and to decrease the risk of adverse drug reactions.

## Impacts on practice

- There are many tools available to assess the use of potentially inappropriate drugs, and different tools might give different prevalences. This is important to be aware of.
- Identification of potentially inappropriate drugs is important, since these drugs have been associated with negative outcomes for the patient. However, as some of the drugs considered inappropriate according to EU (7)-PIM in this study are used as first-hand choices in clinical practice in Sweden, the pertinence of these criteria in clinical practice can be questioned.

## Introduction

Multiple morbidity in older patients contributes to an increased use of medications, which increases the risk of interactions both between medications and between medications and diseases. This makes the treatment of older patients more complicated and the effects of medication treatments more difficult to predict and evaluate [1, 2]. Furthermore, the changes in pharmacokinetic and pharmacodynamic functions that follow with ageing, for example impaired renal function, increase the risk of adverse drug effects [3]. Some of the medications used among older patients are known to be potentially inappropriate medications (PIMs), defined as medications for which the risks outweigh the benefits [4]. The use of PIMs among older patients is a worldwide problem, and many studies have found a high prevalence of PIMs. For example, a register-based study from Sweden where a prevalence of 19% was found when the Swedish quality indicators were applied. In this study, patients 65 years and older in nursing homes and in hospital were included [5]. Another study found that according to the European Union (EU) (7)-PIM list, 41% in-hospital patients had one or more PIM prescribed [6]. Outside Sweden, one study using Beers criteria found a prevalence of 58% among older in-hospital patients in Italy, and another study performed among old patients discharged from a hospital in Croatia found PIMs in 184 of 267 patients (67%) when the EU (7)-PIM list was applied [4, 7]. The use of PIMs has been associated with negative outcomes, for example, increased risk of hospitalisation [8, 9].

To be able to describe drug use in terms of quality, and to be able to assess and correct older patients’ medication regimens, tools or criteria to evaluate the appropriateness of prescriptions have been developed [10]. Criteria can be classified as implicit or explicit criteria. Implicit criteria (or patient-specific criteria) rely on expert professional judgement and focus on the patient, addressing the entire medication regimen. Explicit criteria on the other hand can be applied with little or no clinical judgement, and the criteria are not patient-specific, i.e. the medications are considered inappropriate regardless of the effects on the individual patient [11]. Explicit criteria might be country specific and might need to be adjusted to country-specific therapeutic traditions [12]. Among of the most commonly used and studied explicit criteria are Beer’s criteria, developed in the US for the evaluation of PIMs among older patients [13–16]. Beside these criteria, several other explicit criteria have been developed in different countries [12]. In Sweden, indicators for evaluating the quality of older patients’ drug therapies were first published in 2004 and updated in 2010 and 2017 [17, 18]. Recently, a European list of PIMs has been developed, the EU (7)-PIM list [19]. The different tools to assess PIMs seem however to have a large variety in methodological aspects and in clinical validation, and prevalence of PIM has significantly varied when different criteria have been applied [12, 20]. This raises questions about the appropriateness, validity and how feasible the different tools are in use. Since both the Swedish quality indicators and the (EU) (7)-PIM list have been developed for use in Sweden/Europe, a comparison between prevalence and type of PIMs using the two tools is warranted. As far as we know, this has so far not been done.

## Aim of the study

The aim of this study was to compare the prevalence of PIMs among older patients admitted to a medical ward using the EU (7)-PIM list and the Swedish quality indicators. Secondary objectives were to investigate factors associated with the use of PIMs using the two tools.

## Ethics approval

The regional ethical review board in Umeå approved this study with Registration No. 2014/322-31Ö. All patients were informed about the study and gave their informed consent.

## Methods

### Population

This was a cross-sectional study and was part of a larger study that investigated the impact of medication reviews performed by clinical pharmacists [21]. The study was conducted at a medical ward at a hospital in Lycksele, a small, sparsely populated area of Northern Sweden with no previous experience of clinical pharmacy. This inland hospital provides healthcare services for around 40,000 patients [21]. Data were collected in September–November 2015 and February–April 2016, i.e. when the clinical pharmacists were present at the ward. Patients 18 years or older and admitted to the medical ward when the clinical pharmacists were working in the ward were invited to participate in the study. Exclusion criteria included patients with dementia, palliative patients, patients who did not speak Swedish, intoxicated patients and patients under the influence of alcohol. In this specific study, patients younger than 65 years were excluded because the EU (7)-PIM list and the Swedish quality indicators have been developed to identify PIMs only among older patients.

### Definitions and data extraction

The medical ward where the medication reviews was performed, contains 18 beds and the treatment covers a wide range of diseases. All demographic and medical data such as sex, age, laboratory values, medications, medical history, living conditions, and whether patients had multidose drug dispensing were collected from the medical records at the time of the patients’ admission to the hospital. All this data were used in order to perform the medication reviews. More details about the medication reviews can be found elsewhere [21]. For this specific study, data regarding age, sex and some of the most common diagnoses were collected from the medical records as well as regularly used medication and doses that the patients used at admission to the hospital ward. Pro re nata medications were not included in the analysis due to lack of information about the patients’ use. Locally administrated medications (topical preparations) were also excluded. PIMs were identified using the Swedish indicators for evaluating the quality of older patients’ drug therapies and the EU (7)-PIM list as described below.

#### The Swedish indicators

The Swedish quality indicators for evaluation of older patients’ drug therapies contain nine different drug-specific indicators, one of them is medications that should be avoided unless there is a special reason for using them, and another is medications for which correct and current indication are of particular importance [18]. Other drug-specific indicators are for example polypharmacy, and medications and renal failure. In this specific study, the indicator medications that should be avoided unless there is a special reason for using them is included. This indicator includes long-acting benzodiazepines (nitrazepam, flunitrazepam, and diazepam), medications with significant anticholinergic effects, and the following substances: tramadol, propiomazine, codeine, and glibenclamide. Non-steroidal anti-inflammatory medications (NSAIDs) (M01A excl. M01AX05) and antipsychotic medications (N05A excl. N05AN) are classified as medications for which correct and current indication are of particular importance according to the Swedish quality indicators. Due to the risk of adverse drug reactions among older patients, these medications are also included in this specific study, and classified in the same way as the others, i.e. these medications should be avoided unless there is a special reason for using them. In total, 68 substances were included in the analysis.

#### The EU (7)-PIM list

The complete EU (7)-PIM list comprises 282 drug substances classified as PIMs [19]. Medications that were defined as treatment duration-dependent PIMs according to the EU (7)-PIM list [PPI (pantoprazole, lansoprazole, omeprazole, esomeprazole, rabeprazole), loperamide, nitrofurantoin, naproxen, ibuprofen, codeine, and risperidone] and regimen-dependent PIMs according to the same list (insulin, sliding scale) were excluded due to a lack of information in the medical records. Medications not approved for the Swedish market were also excluded. In this study, a total of 137 substances were selected for the analysis (“Appendix”).

### Data analysis

A simple logistic regression analysis was conducted to investigate the association between patients with PIMs and several factors. These factors included continuous factors; age and number of medications at admission, and categorical factors; sex and certain diagnoses (arrhythmias, cancer, chronic respiratory disease, diabetes mellitus, hypertension, heart failure, stroke/TIA). Results are presented as odd ratios (ORs) with 95% confidence intervals (CIs). All statistical calculations were performed using SPSS version 25, and a *p* value of < 0.05 was considered statistically significant.

## Results

Of 103 patients included in the main study, 10 patients < 65 years were excluded in this specific study, leaving 93 patients’ data to be analysed. The age was 79.5 ± 8.2 years (mean ± SD), and 51 (54.8%) were women. The number of regularly prescribed medications at admission was 8.2 ± 3.6 (mean ± SD). Furthermore, 46 (49.5%) patients had hypertension and 26 (28.0%) patients had arrhythmias (Table 1).

**Table 1:** Characteristics of the study population

Characteristics	Total (n = 93)
Age (years), mean ± SD	79.5 ± 8.2
Women, n (%)	51 (54.8)
Number of regularly prescribed medications at admission, mean ± SD	8.2 ± 3.6
Diseases	
Arrhythmias, n (%)	26 (28.0)
Cancer, n (%)	21 (22.6)
Chronic respiratory disease, n (%)	15 (16.1)
Diabetes mellitus, n (%)	17 (18.3)
Hypertension, n (%)	46 (49.5)
Heart failure, n (%)	22 (23.7)
Stroke/TIA, n (%)	10 (10.8)

*SD* standard deviation, *TIA* transient ischemic attack

According to the Swedish quality indicators, 17 (18.3%) patients in the study sample had one PIM. No patient had more than one PIM prescribed concomitantly. The most commonly represented PIM class among the identified prescriptions according to the Swedish quality indicators (n = 17) was NSAIDs [n = 5 (29.4%)] (Table 2). The most commonly involved PIMs were diclofenac [n = 4 (23.5%)] and tramadol [n = 3 (17.6%)].

**Table 2:** Prescribing frequency for each identified PIM according to the Swedish quality indicators and the EU (7)-PIM list

ATC code	Drug class/name (ATC code)	Prescriptions, n (col%) Swedish quality indicators	Prescriptions, n (col%) EU (7)-PIM list
A03F	Medications for functional gastrointestinal disorder—propulsives	n/a	1 (1.6%)
	Metoclopramide (A03FA01)	n/a	1 (1.6%)
A06A	Laxatives	n/a	3 (4.7%)
	Sodium picosulfate (A06AB08)	n/a	3 (4.7%)
A 10	Blood glucose lowering medications, excl. insulins	n/a	2 (3.1%)
	Glibenclamide (A10BB01)	1 (5.9%)	1 (1.6%)
	Glipizide (A10BB07)	n/a	1 (1.6%)
B01A	Antithrombotic agents	n/a	11 (17.2%)
	Rivaroxaban (B01AF01)	n/a	1 (1.6%)
	Apixaban (B01AF02)	n/a	10 (15.6%)
C01	Cardiac therapy	n/a	8 (12.5%)
	Digoxin (C01AA05)	n/a	4 (6.3%)
	Amiodarone (C01BD01)	n/a	4 (6.3%)
C02	Antihypertensive therapy	n/a	1 (1.6%)
	Doxazosin (C02CA04)	n/a	1 (1.6%)
C03D	Diuretics, potassium-sparing agents	n/a	5 (7.8%)
	Spironolactone (> 25 mg/days)	n/a	5 (7.8%)
C07	Betablocking agents	n/a	1 (1.6%)
	Sotalol (C07AA07)	n/a	1 (1.6%)
C08	Calcium channel blockers	n/a	1 (1.6%)
	Diltiazem (C08DB01)	n/a	1 (1.6%)
G03C	Oestrogens (oral)	n/a	2 (3.1%)
	Estradiol (G03CA03)	n/a	1 (1.6%)
	Estriol (G03CA04)	n/a	1 (1.6%)
G04	Other urologicals, incl. antispasmodic medications	n/a	2 (3.1%)
	Tolterodine (G04BD07)^a^	1 (5.9%)	1 (1.6%)
	Solifenacin (G04BD08)^a^	1 (5.9%)	1 (1.6%)
M01A	NSAID	5 (29.4%)	4 (6.3%)
	Diclofenac (M01AB05)	4 (23.5%)	4 (6.3%)
	Naproxen (M01AE02)	1 (5.9%)	n/a
N02	Analgesics—opioids	n/a	3 (4.7%)
	Codeine (N02AJ06)	1 (5.9%)	n/a
	Tramadol (N02AX02)	3 (17.6%)	3 (4.7%)
N03A	Antiepileptics	n/a	1 (1.6%)
	Carbamazepine (N03AF01)	n/a	1 (1.6%)
N04	Antiparkinson medications	n/a	2 (3.1%)
	Pramipexole (N04BC05)	n/a	2 (3.1%)
N05A	Antipsychotics	1 (5.9%)	1 (1.6%)
	Flupentixol (N05AF01)	1 (5.9%)	1 (1.6%)
N05B	Anxiolytic medications	n/a	2 (3.1%)
	Hydroxyzine (N05BB01)^a^	1 (5.9%)	1 (1.6%)
	Diazepam (N05BA01)	1 (5.9%)	1 (1.6%)
N05C	Hypnotics and sedatives	n/a	12 (18.8%)
	Zopiclone (N05CF01) > 3.75 mg/days	n/a	9 (14.1%)
	Zolpidem (N05CF02) > 5 mg/days	n/a	1 (1.6%)
	Clomethiazole (N05CM02)	n/a	1 (1.6%)
	Propiomazine (N05CM06)	1 (5.9%)	1 (1.6%)
N06A	Antidepressants	n/a	2 (3.1%)
	Amitriptyline (N06AA09)^a^	1 (5.9%)	1 (1.6%)
	Venlafaxine (N06AX16)	n/a	1 (1.6%)

^a^Anticholinergic medications according to Swedish quality indicators [in total 4 (23.5%) prescriptions]

According to the EU (7)-PIMs list, 42 (45.2%) patients had one or more PIMs, of whom 25 (26.9%) had one PIM, 13 (14.0%) had two PIMs, 3 (3.2%) had three PIMs, and 1 (1.1%) had four PIMs. The three most commonly represented PIM classes among the identified prescriptions (n = 64) were hypnotics and sedatives [n = 12 (18.8%)], antithrombotic agents [n = 11 (17.2%)], and cardiac therapy [n = 8 (12.5%)]. The most commonly involved PIMs were apixaban [n = 10 (15.6%)] and zopiclone [n = 9 (14.1%)] (Table 2).

No significant associations between having a PIM according to the Swedish quality indicators and diseases, sex, age, or number of medications at admission were found in the regression analysis (Table 3).

**Table 3:** Comparison between patients with and without PIMs using the Swedish quality indicators as the identification tool

Characteristic of study sample	PIM	No PIM	Simple OR (95% CI)
Cases, n	17	76	
Sex, n (%)			
Female	10 (58.8)	41 (53.9)	1.22 (0.42–3.54)
Age (years), mean ± SD	78.3 ± 8.2	79.7 ± 8.3	0.94 (0.92–1.05)
Number of medications at admission, mean ± SD	9.7 ± 2.6	7.8 ± 3.7	1.16 (1.00–1.35)
Diseases			
Arrhythmias, n (%)	2 (11.8)	24 (31.6)	0.30 (0.06–1.37)
Cancer, n (%)	3 (17.6)	18 (23.7)	0.69 (0.18–2.68)
Chronic respiratory disease, n (%)	5 (29.4)	10 (13.2)	2.75 (0.80–9.48)
Diabetes mellitus, n (%)	6 (35.3)	11 (14.5)	3.22 (0.99–10.50)
Hypertension, n (%)	12 (70.6)	34 (44.7)	2.97 (0.95–9.24)
Heart failure, n (%)	4 (23.5)	18 (23.7)	0.99 (0.29–3.42)
Stroke/TIA, n (%)	2 (11.8)	8 (10.5)	1.13 (0.22–5.89)

No significant variables were found in the simple model, so no multiple analysis was performed

*CI* confidence interval, *OR* odds ratio, *SD* standard deviation, *TIA* transient ischemic attack

No significant associations between age, sex, diseases, number of medications at admission, and having PIMs according to the EU (7)-PIM list were found (Table 4).

**Table 4:** Comparison between patients with and without PIMs using the EU (7)-PIM list as the identification tool

Characteristic of study sample	PIM	No PIM	Simple OR (95% CI)
Cases, n	42	51	
Sex, n (%)			
Female, n (%)	22 (52.4)	29 (56.9)	0.83 (0.37–1.90)
Age (years), mean ± SD	80.3 ± 7.8	78.8 ± 8.6	1.02 (0.97–1.08)
Number of medication at admission, mean ± SD	8.2 ± 3.1	8.2 ± 3.9	1.00 (0.89–1.13)
Diagnosis			
Arrhythmias, n (%)	14 (33.3)	12 (23.5)	1.63 (0.65–4.04)
Cancer, n (%)	10 (23.8)	11 (21.6)	1.14 (0.43–3.01)
Chronic respiratory disease, n (%)	9 (21.4)	6 (11.8)	2.05 (0.66–6.31)
Diabetes mellitus, n (%)	7 (16.7)	10 (19.6)	0.82 (0.80–2.38)
Hypertension, n (%)	21 (50.0)	25 (49.0)	1.04 (0.46–2.35)
Heart failure, n (%)	10 (23.8)	12 (23.5)	1.02 (0.39–2.65)
Stroke/TIA, n (%)	3 (7.1)	7 (13.7)	0.48 (0.12–2.00)

No significant variables were found in the simple model, so no multiple analysis was performed

*CI* confidence interval, *OR* odds ratio, *SD* standard deviation, *TIA* transient ischemic attack

## Discussion

The main finding of this study was that 18% of the study population was taking one PIM according to the Swedish quality indicators, and 45% were taking one or more according to the EU (7)-PIM list. Furthermore, according to the Swedish quality indicators, the most common PIMs were diclofenac and tramadol, while according to the EU (7)-PIM list the most common PIMs were apixaban and zopiclone.

The prevalence of PIMs according to the Swedish quality indicators (18%) is very similar to a previous register study in Sweden (19%) where the same tool was applied, although not exactly the same indicators [5]. When applying the Swedish quality indicators, the most frequently occurring PIMs in the present study were diclofenac, tramadol, and anticholinergic medications. The use of tramadol in older patients increases the risk for nausea, fatigue, dizziness, and confusion and therefore should be prescribed carefully to this patient group [18]. Further, the use of NSAIDs is associated with risks of gastrointestinal bleeding, acute renal failure, and impaired heart failure [22, 23]. Anticholinergic medications such as hydroxyzine increase the risk of constipation and urinary retention as well as confusion and should be used with caution [24]. Notably, the use of tramadol, NSAIDs, and anticholinergic medications decreased between 2007 and 2013, perhaps at least partly due to medication reviews performed in the county of Västerbotten [25]. The prevalence of PIMs among older patients according to the EU (7)-PIM list is also in line with previous studies using the same identification tool, a prevalence between 41% and 67% has been reported [6, 7, 26–28]. According to the EU (7)-PIM list, apixaban was the most commonly prescribed PIM in the present study. Current recommendations published in Sweden in 2017 state that apixaban is recommended as one of the first-line treatment choices for arrhythmias [29]. The recommendation states that apixaban causes fewer haemorrhagic strokes, fewer severe bleedings, and a lower mortality compared to warfarin. Nevertheless, there is limited experience regarding the use of apixaban in older patients, and the drug presents an increased risk of bleeding events. It is therefore important to continuously evaluate the use of the drug and adjust the dosage if necessary [29]. Further, zopiclone at doses > 3.75 mg was the second most common PIM according to the EU (7)-PIM list. In Sweden, zopiclone is the first-line sedative recommendation for older patients in Sweden, with a maximum daily dose of 7.5 mg among this population (although 5 mg often is considered enough) [18]. Still, falls and impaired cognitive function are possible adverse drug reactions to zopiclone, which is why it should be used with caution [18].

In order to improve the use of medication among older patients and to minimize adverse drug effects, the medications prescribed to this patient population must be continuously evaluated. Tools and criteria might therefore be used to assess the appropriateness of a medication, and it is important that these tools and criteria are reliable when it comes to detecting PIMs. Consequently, it is of interest to compare the prevalence obtained by using different tools. When considering the different results in this study using the two different tools, it is important to note the heterogeneity in the lists of medications between the tools. The EU (7)-PIM list is deemed to be a sensitive tool, which might explain the high prevalence of PIMs when the suggested criteria are applied [19]. In the present study, 68 substances were classified as PIMs according to the Swedish quality indicators (including NSAIDs and antipsychotics), while 137 substances were classified as such according to the EU (7)-PIM list. Furthermore, the EU(7)-PIM list recommends lower maximum doses in some cases compared to current Swedish guidelines [18]. To some extent, the higher prevalence of PIMs when using the EU (7)-PIM list is due to the fact that some medications on that list, such as zopiclone and apixaban, are recommended as first-line treatments according to the Swedish guidelines as discussed above. If apixaban and zopiclone were to be excluded, the prevalence of PIMs would decrease from 45 to 25% according to the EU (7)-PIM list. In accordance to the results of the present study, two previous studies comparing EU (7)-PIM and national PIM criteria found that the prevalence of PIMs according to EU (7)-PIM were higher than according to the national lists [7, 27]. Altogether, this raises the question about the pertinence of explicit criteria. Identifying PIMs are important in order to reduce drug-related problems among old patients, but of course, in some patients, prescription of these medications might have been medically well motivated and valid. In practice, these criteria should always be the used in consideration with an individual medical judgment.

There were no significant associations in the simple analysis between sex, age, higher number of medications, or different diseases and having PIMs according to the Swedish quality indicators. This is in contrast with the findings of a nationwide, cross-sectional, register-based study using the criteria from the Swedish quality indicators, where significant associations between women, age, and a higher number of medications and having PIMs were found [5]. Further, no significant associations with the factors mentioned above and PIMs in the simple analysis were found according to the EU (7)-PIM list. Perhaps an association with the use of PIMs would have been expected for arrhythmias due to the high prevalence of apixaban. In previous research, the observed associated factors varied from study to study, and this might be the result of different study locations and study samples even though the same identification tool was used [6, 26, 27]. However, the reason for the lack of significant associations in the present study might be due to the small study sample.

Strengths of the study are the fact that the medication records used are a reliable source, and as far as we are aware the present study is also the first study that compares the prevalence of PIMs using both the EU (7)-PIM list and the Swedish quality indicators.

There are some limitations to consider with the present study. First and most important, the number of patients included was limited. Thus, the representativeness of the study population is low and the results should be interpreted carefully because there is risk of bias and chance findings. Also, the chance to find statistically significant relationships is very low due to the limited number of observations, and the results should be interpreted with caution for that reason as well.

A new version of the Swedish quality indicator was used in the analysis, a version that was not published when the data were collected, and this has to be taken into account when interpreting the results. Among substances prevalent in the study population, glibenclamide and codeine were not listed in the version from 2010, the list that was valid in 2015–2016 when data was collected. Also, the use of antipsychotic medications and NSAIDs were included in the quality indicators, although the indications for prescribing were not assessed. Further, a total of 282 substances are identified as PIMs according to the EU (7)-PIM list, but only 137 substances were evaluated in this present study because many of the medications are not approved for use in Sweden. The duration and regimen-dependent PIMs and pro re nata medications were also excluded, which might lower the prevalence of PIMs among the study population according to the EU (7)-PIM list.

## Conclusion

The prevalence of PIMs according to the Swedish quality indicators is relatively low in comparison with the EU (7)-PIM list. No statistically significant associated factors with PIMs were found with either list, possibly due to the small study sample. Although different evaluation tools might give inconclusive results, it is still important to continuously evaluate the use and need for PIMs in older patients in order to decrease the risk of adverse drug events.

## Appendix

**Table:**

	Comment	ATC code
*A*		
Acarbose		A10BF01
Acetylsalicylic acid	> 325 mg	N02BA01
Alimemazine		R06AD01
Almotriptan		N02CC05
Alprazolam		N05BA12
Aluminium-containing antacids		A02AD01
Amfebutamone		N06AX12
Amiodarone		C01BD01
Amitriptyline		N06AA09
Apixaban		B01AF02
Aripiprazole		N05AX12
Atropine		A03BA01
*B*		
Baclofen		M03BX01
Biperiden		N04AA02
Bromocriptine		N04BC01
*C*		
Cabergoline		N04BC06
Carbamazepine		N03AF01
Celecoxib		M01AH01
Chlorprothixene		N05AF03
Clemastine		R06AA04
Clomethiazole		N05CM02
Clomipramine		N06AA04
Clonazepam		N03AE01
Clonidine		C02AC01
Clozapine		N05AH02
*D*		
Dabigatran		B01AE07
Darifenacin		G04BD10
Dexketoprofen		M01AE17
Diazepam		N05BA01
Diclofenac (oral)		M01AB05
Digoxin		C01AA05
Diltiazem		C08DB01
Dimenhydrinate		R06AA02
Dipyridamole		B01AC07
Disopyramide		C01BA03
Doxazosin		C02CA04
Dronedarone		C01BD07
Droperidol		N05AD08
*E*		
Ebastine		R06AX22
Eletriptan		N02CC06
Estradiol	Oral	G03CA03
Estriol	Oral	G03CA04
Etoricoxib		M01AH05
*F*		
Famotidine		A02BA03
Ferrous sulfate	> 325 mg/d	B03AA01/07
Fesoterodine		G04BD11
Flecainide		C01BC04
Flunitrazepam		N05CD03
Fluoxetine		N06AB03
Flupentixol		N05AF01
Fluphenazine		N05AB02
Fluvoxamine		N06AB08
Frovatriptan		N02CC07
*G*		
Glibenclamide		A10BB01
Glimepiride		A10BB12
Glipizide		A10BB07
*H*		
Haloperidol	> 2 mg single dose or > 5 mg/d	N05AD01
Hydralazine		C02DB02
Hydroxyzine		N05BB01
Hyoscine		A04AD01
Hyoscyamine		A03BA03
*I*		
Ivabradine		C01EB17
*K*		
Ketoprofen		M01AE03
Ketorolac		M01AB15
*L*		
Labetalol		C07AG01
Levomepromazine		N05AA02
Lithium		N05AN01
Lorazepam	> 1 mg/d	N05BA06
*M*		
Maprotiline		N06AA21
Meclozine		R06AE05
Meloxicam		M01AC06
Methadone		N07BC02
Methylphenidate		N06BA04
Metoclopramide		A03FA01
Midazolam		N05CD08
Moxonidine		C02AC05
*N*		
Nabumetone		M01AX01
Naratriptan		N02CC02
Nifedipine	Non-sustained-release/sustained-release	C08CA05
Nitrazepam		N05CD02
Nortriptyline		N06AA10
*O*		
Olanzapine	> 10 mg/d	N05AH03
Orphenadrine		M03BC01
Oxazepam	> 60 mg/d	N05BA04
Oxybutynin	Non-sustained-release/sustained-release	G04BD04
*P*		
Paroxetine		N06AB05
Perphenazine		N05AB03
Pethidine		N02AB02
Phenobarbital		N03AA02
Phenylpropanolamine		R01BA01
Phenytoin		N03AB02
Pindolol		C07AA03
Pioglitazone		A10BG03
Piracetam		N06BX03
Piroxicam		M01AC01
Pramipexole		N04BC05
Prasugrel		B01AC22
Promethazine		R06AD02
Propafenone		C01BC03
Propiomazine		N05CM06
Propranolol		C07AA05
Prucalopride		A06AX05
*R*		
Racecadotril		A07XA04
Ranitidine		A02BA02
Reboxetine		N06AX18
Rivaroxaban		B01AF01
Rizatriptan		N02CC04
Ropinirole		N04BC04
Rotigotine		N04BC09
*S*		
Selegiline		N04BD01
Senna glycosides		A06AB06
Sertindole		N05AE03
Sitagliptin		A10BH01
Sodium picosulfate		A06AB08
Solifenacin		G04BD08
Sotalol		C07AA07
Spironolactone	> 25 mg/d	C03DA01
Strontium ranelate		M05BX03
Sumatriptan		N02CC01
*T*		
Terazosin		G04CA03
Terbutaline	Oral	R03CC03
Theophylline		R03DA04
Tibolone		G03CX01
Tolterodine	Non-sustained-release/sustained-release	G04BD07
Topiramate		N03AX11
Tramadol	Non-sustained-release/sustained-release	N02AX02
Triazolam		N05CD05
Trihexyphenidyl		N04AA01
*V*		
Venlafaxine		N06AX16
Verapamil		C08DA01
Vildagliptin		A10BH02
*Z*		
Zaleplon	> 5 mg/d	N05CF03
Ziprasidone		N05AE04
Zolmitriptan		N02CC03
Zolpidem	> 5 mg/d	N05CF02
Zopiclone	> 3.75 mg/d	N05CF01
Zuclopenthixol		N05AF05

*d* day, *IR* immediate release, *SR* slow release
